# Association of Dietary Intake and Biomarker Levels of Arsenic, Cadmium, Lead, and Mercury among Asian Populations in the United States: NHANES 2011–2012

**DOI:** 10.1289/EHP28

**Published:** 2016-09-02

**Authors:** Hiroshi Awata, Stephen Linder, Laura E. Mitchell, George L. Delclos

**Affiliations:** 1 University of Texas Health Science Center at Houston School of Public Health, Houston, Texas, USA; 2 CH2M HILL, Inc., Houston, Texas, USA

## Abstract

**Background:**

We have recently shown that biomarker levels of selected metals are higher in Asians than in other U.S. ethnic groups, with important differences within selected Asian subgroups. Much of this difference may be dietary in origin; however, this is not well established.

**Objective:**

We evaluated dietary intake of toxic metals as a source of increased biomarker levels of metals among U.S. Asians.

**Methods:**

We estimated daily food consumption and dietary intake of arsenic, cadmium, lead, and mercury by combining 24-hr dietary intake recall data from the 2011–2012 National Health and Nutrition Examination Survey (NHANES) with data from the USDA Food Composition Intake Database and FDA Total Dietary Study. We analyzed associations between dietary metal intake and biomarker levels of the metals using linear regression. Further, estimated food consumption and metal intake levels were compared between Asians and other racial/ethnic groups (white, black, Mexican American, and other Hispanic) and within three Asian subgroups (Chinese, Indian Asian, and other Asians).

**Results:**

Significant associations (*p* < 0.05) were found between biomarker levels and estimated dietary metal intake for total and inorganic arsenic and mercury among Asians. Asians had the highest daily fish and rice consumption across the racial/ethnic groups. Fish was the major contributor to dietary mercury and total arsenic intake, whereas rice was the major contributor to inorganic arsenic dietary intake. Fish consumption across the Asian subgroups varied, with Asian Indians having lower fish consumption than the other Asian subgroups. Rice consumption was similar across the Asian subgroups.

**Conclusions:**

We confirmed that estimated dietary intake of arsenic (total and inorganic) and mercury is significantly associated with their corresponding biomarkers in U.S. Asians, using nationally representative data. In contrast, estimated dietary intake of cadmium and lead were not significantly associated with their corresponding biomarker levels in U.S. Asians.

**Citation:**

Awata H, Linder S, Mitchell LE, Delclos GL. 2017. Association of dietary intake and biomarker levels of arsenic, cadmium, lead, and mercury among Asian populations in the United States: NHANES 2011–2012. Environ Health Perspect 125:314–323; http://dx.doi.org/10.1289/EHP28

## Introduction

Biomarker levels of toxic metals/metalloids (hereafter, simply referred to as “metals”), such as arsenic, cadmium, lead, and mercury, are higher among the Asian population than other racial/ethnic groups in the United States. A publication from the Centers for Disease Control and Prevention (CDC) reported that Asians had biomarker levels of these metals up to four times higher than other racial/ethnic groups (CDC 2014). For instance, the geometric mean blood mercury (total) level among Asians was 1.86 μg/L as compared with 0.48 μg/L in Mexican Americans.

Arsenic, cadmium, lead, and mercury are well known toxic environmental contaminants. U.S. and international environmental and public health agencies have classified inorganic arsenic and cadmium as human carcinogens (IARC 2013; U.S. EPA 2015). Further, exposure to these metals has been associated with a number of adverse health effects, including developmental and nervous system damage (ATSDR 1999, 2007a, 2007b, 2012). Hence, these elevated biomarker levels reflect a potentially increased health risk among the Asian population.

Food consumption is considered one of the predominant exposure pathways of these toxic metals. These metals are bioaccumulative and ubiquitous in the environment. Although mitigation efforts in the United States over the past few decades have largely succeeded in controlling their release into the environment, they are still detectable in many foods. Several of these foods are consumed by Asian Americans in large amounts. For instance, elevated levels of mercury and arsenic (total) in seafood and arsenic (inorganic) in grains (e.g., rice) have been reported (FDA 2014a, 2014b). These foods are staples of the Asian diet. However, studies characterizing dietary intake levels of these metals among the Asian populations (i.e., the populations that appear to be at highest risk of exposure based on biomarker studies) in the United States were conducted mostly in cohorts from geographic areas with high Asian populations. Consequently, our understanding of dietary exposure characteristics of Asians on a national scale is fairly limited.

To fill this gap, we evaluated dietary intake of these metals in the United States, based on nationally representative data. We evaluated the association between dietary metal intake and biomarker levels across various racial/ethnic groups (Asian, white, black, Mexican American, and other Hispanic). In addition, because Asians in the United States comprise several different ethnic subpopulations that may have different dietary patterns, we evaluated these associations across two major Asian subgroups (Chinese and Asian Indian). Finally, we examined variations in food consumption and dietary metal intake across these same groups and subgroups to identify the foods that contribute most to their overall dietary metal intake.

## Methods

### Study Population

The National Health and Nutrition Examination Survey (NHANES) was used as the primary data source for this study. The NHANES is a national population-based survey program assessing the health and nutritional status of the civilian noninstitutionalized general U.S. population. Health and nutrition data are collected each year from approximately 5,000 survey participants, selected using a complex, multistage, probability sampling design (Johnson et al. 2014). The multistage sampling procedure is comprised of four stages of geographical unit selection. It starts with a selection of the primary sampling units (typically at the county level) and then selects smaller geographical units (city blocks and then households) within the units at each subsequent stage. At the final stage, more than one individual is often drawn from a single household. The NHANES data are released every 2 years.

This study used the data from the most recent data cycle (2011–2012) because it was the first NHANES cycle to oversample Asians. The non-Hispanic Asian category includes individuals with self-reported origins in the Far East Asia, Southeast Asia, or South Asia (the Indian subcontinent) (NCHS 2013). We further subcategorized Asians into the two largest Asian subgroups: Chinese (Chinese and Taiwanese) and Asian Indian (Asian Indian, Bengalese, Bharat, Dravidian, East Indian, and Goanese), and combined all the remaining Asians into an “Other Asian” subgroup, to investigate variations across Asian subgroups. No specific subgroups within the Asian population were oversampled in NHANES 2011–2012.

Sociodemographic, dietary, and biomarker data from the NHANES 2011–2012 cycle are publicly available and were obtained directly from the CDC website. Because access to data on Asian ancestry and geographical information is restricted, analyses of these variables were conducted at the CDC Research Data Center (RDC) in Atlanta, Georgia (NCHS-RDC 2012) following review and approval by the NCHS Research Ethics Review Board.

### Estimation of Food Consumption and Dietary Metal Intake

We used three data sets to estimate dietary metal intake. A brief description of each data set is provided below and is summarized in Table 1.

**Table 1:** Summary of data sources used in the estimation of dietary metal intake.

Data type	Source	Description	Major limitation
Consumption data	USDA What We Eat in America Study (as part of 2010–2011 NHANES dietary Data) (USDA 2014)	24-hr diet recall data collected on two nonconsecutive survey days. Food items are recorded “as consumed” (e.g., lasagna).	Snapshot of food consumption and may not represent long-term food consumption characteristics. Subject to recall bias.
Composition data	USDA/EPA Food Commodity Intake Database 2005–2010 (U.S. EPA 2014)	Recipe file including amount of each “food commodity” included in 100 g of “as consumed food recorded in NHANES.”	Recipe does not explain individual variations in cooking methods or food preparation.
Chemical data	FDA TDS 2006–2011 (FDA 2013)	Chemical residue data in food collected using “market basket” approach. Samples are collected in three cities from each of four regions of the United States.	The estimation of dietary metal intake used single representative concentrations (mean), which do not account for variations in chemical concentrations across different food types, geographical locations of cultivation, growing methods, cooking/preparation, among others. Lack of the data for food that may be important for metal intake (e.g., seaweed). Uncertainties associated with non-detected results.
	Schoof et al. (1999)	Chemical residue data in 40 foods collected using a modified “market basket” approach (inorganic arsenic only).	Data collection was performed in an older time period (1997).

***Consumption data.*** The NHANES food consumption data were used to estimate the types and amounts of food consumed by study participants (USDA 2014). These data were collected using an interviewer-administered questionnaire that included a 24-hr dietary recall instrument. The interview was administered on two nonconsecutive survey days, 3–10 days apart. During the interview, food items were recorded “as consumed” (e.g., meat lasagna), rather than on an individual food component basis (e.g., tomato). We limited our analyses to data from individuals with body weight and food-consumption data available for days 1 and 2. Specific information about collection and processing of the food consumption data is provided online (USDA 2014).

***Composition data.*** The composition of each food item was determined using the Food Commodity Intake Database (FCID) created by the U.S. Environmental Protection Agency (U.S. EPA) and U.S. Department of Agriculture (USDA) (U.S. EPA 2014). This database provides the amount of each individual food ingredient, hereafter referred to as “food commodity,” included in 100 g of each food reported specifically in NHANES. For instance, the FCID food commodities in 100 g of “meat lasagna” include 20.9 g of tomato, 16.2 g of wheat flour, and 7.9 g of beef.

The most recent FCID (FCID 2005–2010) has been updated to the previous NHANES data cycle (2009–2010) but does not include food items added to the 2011–2012 NHANES dietary data. Thus, we identified food items in the current FCID that most closely represent the new food items in terms of food description and composition (U.S. EPA 2014) and used their composition data for these newly added food items. We also used the USDA’s cross-reference information—which presents the changes in the food coding due to expansion, consolidation, and renumbering of coding system between current and previous NHANES data cycles—for this selection process (USDA 2015). For a small number of the food items (< 1% of food items reported in the dietary consumption data) for which a representative food item was not identified, new composition data were created based on the existing data for similar food items and/or the USDA’s Food and Nutrient Database for Dietary Studies (food composition data for nutritional studies) (USDA 2015). We assigned no composition data to food items that were not of interest to the present study (e.g., water, energy and alcoholic drinks, condiments).

***Chemical data.*** The U.S. Federal Drug Administration (FDA) Total Dietary Study (TDS) (FDA 2013) was used as our main source of chemical concentration data. The TDS is a continuous food-safety monitoring program, in which food samples are collected using an approach called “market basket.” The data are collected in three cities from each of four regions of the nation, with one region sampled each quarter (spring in the South, summer in the Northeast, autumn in the North Central region, and winter in the West) (FDA 2015b). Samples of food, collected directly from retail stores and fast-food restaurants in each region, are compiled to create a market basket representing the average U.S. diet.

For this study we used 2006–2011 TDS data. For food commodities that were not included in the 2006–2011 TDS data set, we used TDS data from previous years (1991–2005). The TDS’s effort to analyze mercury in food is targeted at fish and other seafood; therefore, mercury data in other food groups are fairly limited. Specific information about laboratory procedures used in the TDS is presented elsewhere (FDA 2015a).

The TDS does not include data on inorganic arsenic. Consequently, data for inorganic arsenic were obtained from Schoof et al. (1999), who applied a modified market basket survey approach and collected inorganic arsenic data from 40 foods that were expected to contribute to at least 90% of dietary inorganic intake in the general U.S. population (Schoof et al. 1999).

Two dietetic specialists linked food commodities between the composition and chemical data. To focus this effort, food commodities consumed in the largest quantities were determined for each of the five NHANES racial/ethnic groups. Food commodities found to make up 95% of the diet for at least one racial/ethnic group were identified as the target food commodities. Initially, each dietitian linked one-half of the target food commodities. These linkages were then reviewed by both dietitians, with the final linkage based on their consensus decision.

***Estimation approach.*** We estimated daily food consumption and dietary metal intake, generally following the approach presented in the study conducted by Yost et al. (2004):

We translated NHANES’s food consumption data, presented “as consumed,” into food commodities using the food composition database.
Total daily consumption of each food commodity was estimated as the sum of all meals (including snacks) in a 24-hr period. The estimated daily food commodity consumption was divided by survey participant’s body weight to obtain body weight-adjusted daily commodity consumption. Food commodity consumption was calculated for both day 1 and day 2, and the 2-day average was used for daily commodity consumption of each survey participant.
We then estimated food-category consumption by summing the calculated commodity-specific food consumption, obtained in step 2, by 14 major food categories (vegetables, fruits, mushroom, nuts, herbs and spices, cereal grains, beef, pork, poultry, other meat, fish, dairy, egg, and oil) and additional subcategories under cereal grains (white rice and brown rice) and fish (freshwater fish, saltwater fish, and shellfish).
Based on the chemical data, we estimated daily dietary metal intake by multiplying the daily commodity consumption, obtained in step 2, by metal concentration in the food commodity. In accordance with the *Fourth National Report on Human Exposure to Environmental Chemicals* (CDC 2014), the level of detection (LOD) divided by the square root of 2 was used as the “fill value” for subjects with non-detected results.
Similar to step 3, we estimated food-category dietary metal intake by summing the calculated commodity-specific, daily, dietary metal intake, obtained in step 4, by 14 major categories and sub-categories.
Last, we estimated total individual dietary metal intake by summing all of the calculated commodity-specific daily dietary metal intakes, obtained in step 4, for each person.

### Biomarker Data

Biomarker data for blood cadmium (B-Cd), blood lead (B-Pb), and blood mercury (B-Hg), as well as urinary total arsenic (U-tAs) and urinary dimethylarsinic acid (U-DMA) were obtained from NHANES. Biomarker data were collected on day 1 of the 2 nonconsecutive food consumption survey days. Blood biomarker samples were collected from survey participants age ≥ 1 year, whereas urinary biomarker samples were collected from a randomly selected one-third of participants, ages ≥ 6 years. Urinary biomarker data were adjusted for creatinine to address the effect of urinary dilution, as computed in the CDC document (CDC 2014). Inorganic arsenic is excreted as inorganic arsenic and methylated metabolites (e.g., monomethylarsonic acid, DMA). Although these methylated arsenic species can also be metabolites of organic arsenic, a sum of these metabolites is commonly used to represent inorganic arsenic exposure (Davis et al. 2012; Wei et al. 2014). As DMA is the predominant detectable inorganic arsenic metabolite, U-DMA concentrations were used in the evaluation of dietary inorganic arsenic intake data. The detection frequency of biomarker data as follows: B-Cd (73%); B-Pb (99%); B-Hg (94%); U-tAs (≥ 96%); U-DMA (≥ 79%). For samples with non-detectable biomarker levels, we used the LOD divided by the square root of 2, as reported in the NHANES data. More information regarding laboratory procedures used for the chemical analyses are presented in the National Center for Environmental Health’s Laboratory Procedure Manuals (NCHS 2011a, 2011b, 2012).

### Statistical Analyses

We performed all statistical analyses using SAS-callable SUDAAN version 11.0.1 (RTI International, Research Triangle Park, NC, USA). SUDAAN was installed as an add-on to SAS software version 9.3 or higher (SAS Institute Inc., Cary, NC, USA). The data were stratified by five NHANES racial/ethnic groups (Asian, white, black, Mexican American, and other Hispanic) and three Asian subgroups (Chinese, Asian Indian, and Other Asian), and the results were presented for each group. All the statistical analyses accounted for the NHANES’s complex sample design and weighting.

***Analysis of associations between biomarker levels and dietary metal intake.*** We evaluated the association between biomarker levels and dietary metal intake using linear regression, with biomarker level as the dependent variable and dietary metal intake level as an independent variable. Both biomarker levels and dietary metal intake data were log-transformed (base of 10) and included in the model as continuous variables. Two models were constructed for the analysis: *a*) bivariate regression model and *b*) multivariate regression model (“full” model) adjusting for all covariates (as indicated below). The association was evaluated for each metal and each of the racial/ethnic groups and subgroups. For the regression analysis, we restricted the data to those individuals who had complete data for all the covariates used in the analysis.

***Descriptive statistics of food consumption and dietary metal intake.*** We computed weighted summary statistics (arithmetic mean, and 50th and 95th percentiles) for body weight (BW)-adjusted food consumption (in units of g-food/kg-BW/day) and BW-adjusted dietary metal intake (in units of μg-metal/kg-BW/day) across 14 major food categories and additional subcategories under cereal grains and fish. Dietary metal intake was calculated for total arsenic, inorganic arsenic, cadmium, lead, and mercury.

We also calculated weighted summary statistics for dietary metal intake within subgroups of each race/ethnicity defined by the following sociodemographic and geographic covariates: sex, age, education, household income, birthplace (United States/non–United States), and urban–rural classification, based on the 2013 NCHS urban–rural classification scheme for counties (Ingram and Franco 2014), and U.S. census region. Because of protections intended to preserve study participants’ confidentiality, we were unable to analyze Asian subgroups using geographical covariates (urbanization and census region). For children (6–19 years), the education level of the household reference person (typically the adult owner or renter of the residence) was used.

We performed pairwise comparisons of arithmetic mean dietary intake for each combination of the five NHANES racial/ethnic groups (e.g., mean intake in Asian females vs. non-Hispanic white females). In addition, we compared each combination of the three Asian subgroups (e.g., mean intake in Chinese females vs. Asian Indian females) to assess variability across the Asian subgroups. Further, differences in arithmetic means of dietary metal intake within each covariate (e.g., Asian males vs. Asian females) were determined using analysis of variance (ANOVA). Statistical significance was determined by *p*-value < 0.05.

## Results

### Sample Characteristics

**Figure 1:**
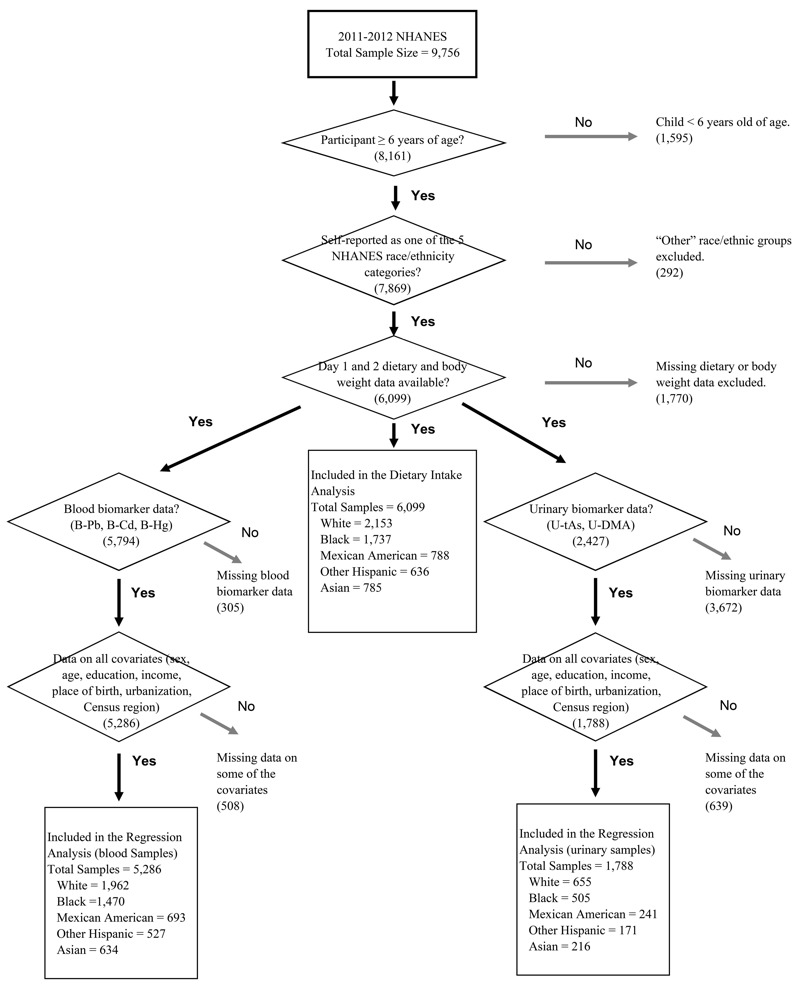
Schematic diagram of inclusion criteria and sample counts. Notes: B-Cd, blood cadmium; B-Hg, blood mercury; B-Pb, blood lead; NHANES, National Health and Nutrition Examination Survey; U-DMA, urinary dimethylarsinic acid; U-tAs, urinary total arsenic.

After excluding children < 6 years of age, “other” race groups, and participants without body weight or dietary data from both interview days, data from 6,099 NHANES participants were included in our analyses (Figure 1). The characteristics of the study population are presented in Table 2.

**Table 2:** Characteristics of study participants [*n* (%) or %] with weighted percentage, NHANES 2011–2012.

Covariates^*a*^	Non-Hispanic Asian [785 (5.2)]	Asian subpopulations^*b*^			Non-Hispanic white [2,153 (66.0)]	Non-Hispanic black [1,737 (12.5)]	Mexican American [788 (9.8)]	Other Hispanic [636 (6.5)]
		Chinese (17.3)	Asian Indian (21.4)	Other Asian (61.3)				
Sex
Male	397 (48.0)	(43.5)	(55.7)	(46.6)	1,082 (49.2)	798 (44.3)	415 (53.0)	300 (48.1)
Female	388 (52.0)	(56.5)	(44.3)	(53.4)	1,071 (50.8)	939 (55.7)	373 (47.0)	336 (51.9)
Age
6–11 years	108 (8.0)	(7.0)	(11.1)	(7.1)	253 (7.3)	289 (10.7)	204 (14.1)	117 (11.2)
12–19 years	143 (10.9)	(11.9)	(8.9)	(11.3)	233 (10.2)	321 (14.7)	176 (18.8)	112 (12.3)
20–39 years	236 (35.3)	(36.7)	(34.6)	(35.2)	530 (26.1)	350 (30.2)	184 (38.1)	121 (34.7)
40–59 years	191 (30.1)	(28.4)	(30.4)	(30.4)	515 (31.8)	402 (29.5)	144 (23.0)	138 (26.6)
≥ 60 years	107 (15.8)	(16.0)	(15.1)	(16.0)	622 (24.7)	375 (15.0)	80 (6.0)	148 (15.2)
Education
< High school (HS)	113 (12.3)	(4.7)	(8.5)	(15.7)	349 (11.8)	343 (19.7)	427 (47.6)	230 (32.3)
HS graduate/GED	90 (11.4)	(10.1)	(9.0)	(12.7)	431 (19.2)	447 (26.7)	172 (21.5)	133 (21.9)
Some college/AA degree	154 (21.2)	(17.7)	(15.1)	(24.3)	717 (32.7)	609 (36.4)	131 (21.7)	152 (28.1)
≥ College graduate	420 (55.1)	(67.6)	(67.5)	(47.3)	638 (36.2)	311 (17.2)	54 (9.2)	107 (17.7)
Household income
< $20,000	77 (11.3)	(7.8)	(7.2)	(13.9)	494 (13.4)	509 (33.6)	212 (27.6)	164 (30.3)
$20,000–< $50,000	226 (34.0)	(33.8)	(29.7)	(35.7)	743 (30.9)	613 (36.6)	375 (44.6)	224 (36.1)
$50,000–< $75,000	82 (12.2)	(9.8)	(21.5)	(9.5)	204 (13.3)	149 (9.0)	71 (11.2)	73 (13.2)
≥ $75,000	317 (42.4)	(48.6)	(41.6)	(40.9)	638 (42.4)	343 (20.7)	96 (16.6)	124 (20.5)
Birthplace
USA	273 (27.8)	(27.1)	(17.4)	(31.6)	2,069 (95.8)	1,604 (93.0)	467 (56.3)	260 (40.3)
Outside USA	512 (72.2)	(72.9)	(82.6)	(68.4)	84 (4.2)	133 (7.0)	321 (43.7)	373 (59.7)
Urbanization^*b*^
Metro center	(57.9)		*_____*^*c*^		(23.7)	(44.3)	(46.9)	(66.8)
Metro fringe	(27.6)				(28.0)	(30.5)	(5.6)	(21.7)
Other	(14.6)				(48.3)	(25.2)	(47.6)	(11.5)
U.S. Census region^*b*^
Northeast	(22.5)		*_____*^*c*^		(14.2)	(11.0)	(5.2)	(33.0)
Midwest	(9.7)				(29.3)	(14.4)	(4.1)	(3.0)
South	(27.6)				(31.2)	(68.2)	(38.9)	(47.3)
West	(40.2)				(25.2)	(6.5)	(51.8)	(16.7)
Abbreviations: AA, Associate in Art degree; GED, General Educational Development. ^***a***^Sample counts and weighted percentage among five NHANES race and ethnic groups and weighted percentage among three Asian subgroups. ^***b***^Raw sample counts are not provided for the restricted data. ^***c***^Because of potential disclosure risk, geographical analysis on Asian subgroups is not included.								

The age distribution of Asians was similar to those of the black and other Hispanic groups. A higher educational status was evident among Asians, compared with other racial/ethnic groups. Household income in Asians closely corresponded to that of whites and was higher than those of other racial/ethnic groups: More than 40% of whites and Asians reported their annual household income was > $75,000. More than 70% of Asians were born outside the United States, whereas the majority of whites and blacks (> 90%) were born in the United States. Geographic variations across the groups were fairly large. Approximately 80% of Asians lived in metro areas, and tended to concentrate mainly in the western region of the United States.

Across the Asian subgroups, the age distribution was generally similar, although the distribution of Asian Indians tended to be slightly shifted to younger ages than those of other two subgroups. Across the Asian subgroups, a higher educational status was observed among Chinese and Asian Indians than the Other Asian subgroup. The percentage of U.S.-born individuals was considerably lower in Asian Indians than in Chinese and Other Asian subgroups. The relative distribution of the Asian subgroups in the study population was similar to that reported in the 2010 Census data (Hoeffel et al. 2012).

### Data Preparation of Dietary Intake

The food consumption data from 6,099 individuals comprised 5,273 unique food items. The food items reported in the consumption data were converted into 386 individual food commodities. Among these items, we identified 123 food commodities as target food commodities based on consumed quantities (i.e., commodities making up 95% of the diet in at least one of the racial/ethnic groups). We were able to assign chemical data to approximately 80% of the target food commodities for their total arsenic, cadmium, and lead content. No chemical data were available for the remaining 20%, but these commodities each comprised < 0.5% of the total food consumed. Because the TDS’s mercury analysis in food is focused on fish and other seafood, we were able to assign mercury content only to 28 out of the 123 target food commodities (roughly 20%).

### Regression Analyses

Table 3 presents the results of linear regression analyses predicting biomarker levels as a function of estimated dietary intake. The results of the bivariate and multivariate models for total and inorganic arsenic were similar. In general, total dietary arsenic intake (DI-tAs) and dietary inorganic arsenic intake (DI-iAs) were significant predictors of U-tAs and U-DMA, respectively (*p* < 0.05). Standardized regression coefficients between total and inorganic arsenic were similar across the racial/ethnic groups and Asian subgroups, ranging from 0.24 to 0.41 (excluding nonsignificant results) for total arsenic, and from 0.26 to 0.59 for the inorganic arsenic model. For both the total and inorganic arsenic models, a higher standardized regression coefficient was observed among the Asian group and Asian subgroups compared with those of other racial/ethnic groups.

**Table 3:** Association between dietary intake of metals and biomarker levels: multiple linear regression results.

Metal	Standardized regression coefficient (SE) (*p*-value)							
	Non-Hispanic Asian 216 (634)^*a*^	Asian subpopulations^*b*^^,^^*c*^			Non-Hispanic white 655 (1,962)^*a*^	Non-Hispanic black 505 (1,470)^*a*^	Mexican American 241 (693)^*a*^	Other Hispanic 171 (527)^*a*^
		Chinese	Asian Indian	Other Asian				
Arsenic, total (urinary)	0.36 (0.04) < 0.001 *R*^2^ = 0.31	0.13 (0.06) 0.31 *R*^2^ = 0.30	0.41 (0.03) < 0.001 *R*^2^ = 0.60	0.34 (0.05) < 0.001 *R*^2^ = 0.36	0.34 (0.07) < 0.001 *R*^2^ = 0.15	0.24 (0.04) < 0.001 *R*^2^ = 0.22	0.26 (0.03) < 0.001 *R*^2^ = 0.07	0.10 (0.06) 0.30 *R*^2^ = 0.18
Arsenic, inorg. (urinary)	0.42 (0.08) < 0.001 *R*^2^ = 0.34	0.59 (0.10) < 0.001 *R*^2^ = 0.48	0.47 (0.11) 0.005 *R*^2^ = 0.56	0.41 (0.11) < 0.001 *R*^2^ = 0.37	0.31 (0.02) < 0.001 *R*^2^ = 0.21	0.26 (0.04) < 0.001 *R*^2^ = 0.24	0.33 (0.05) < 0.001 *R*^2^ = 0.20	0.41 (0.08) < 0.001 *R*^2^ = 0.35
Cadmium (blood)	–0.04 (0.06) 0.36 *R*^2^ = 0.39	0.03 (0.04) 0.47 *R*^2^ = 0.47	–0.11 (0.12) 0.33 *R*^2^ = 0.32	–0.04 (0.06) 0.39 *R*^2^ = 0.46	–0.03 (0.04) 0.28 *R*^2^ = 0.23	0.05 (0.04) 0.13 *R*^2^ = 0.28	0.01 (0.08) 0.90 *R*^2^ = 0.33	0.05 (0.05) 0.23 *R*^2^ = 0.27
Lead (blood)	–0.02 (0.05) 0.61 *R*^2^ = 0.343	–0.10 (0.14) 0.28 *R*^2^ = 0.41	0.02 (0.20) 0.90 *R*^2^ = 0.38	–0.05 (0.07) 0.37 *R*^2^ = 0.28	0.04 (0.06) 0.34 *R*^2^ = 0.38	0.04 (0.05) 0.20 *R*^2^ = 0.41	0.14 (0.06) 0.004 *R*^2^ = 0.36	0.04 (0.08) 0.50 *R*^2^ = 0.34
Mercury (blood)	0.12 (0.05) 0.002 *R*^2^ = 0.31	0.27 (0.11) 0.01 *R*^2^ = 0.45	–0.05 (0.12) 0.56 *R*^2^ = 0.32	0.18 (0.09) 0.02 *R*^2^ = 0.28	0.17 (0.07) 0.003 *R*^2^ = 0.33	0.17 (0.03) < 0.001 *R*^2^ = 0.26	0.14 (0.03) < 0.001 *R*^2^ = 0.26	0.10 (0.05) 0.03 *R*^2^ = 0.32
Standardized regression coefficient presents 1 standard deviation increase in log-transformed biomarker levels associated with 1 standard deviation increase in log-transformed dietary metal intake. ^***a***^Sample counts of urinary biomarker data (sample counts of blood biomarker data). ^***b***^Raw sample counts are not provided for the restricted data. Multiple linear regression model was adjusted for sex, age, education, income, and birth place, urbanization, and census region. ^***c***^Because of potential disclosure risk, geographical covariates (urbanization and census region) were not included in multivariate model for Asian subgroups.								

In multivariate models, dietary cadmium intake (DI-Cd) was not a significant predictor of B-Cd levels in either the main racial/ethnic groups or the Asian subgroups. A significant correlation between B-Pb levels and dietary lead intake (DI-Pb) was found only among Mexican Americans.

Dietary mercury intake (DI-Hg), on the other hand, was a significant predictor of B-Hg levels among all racial/ethnic groups and subgroups, except Asian Indians. The Chinese subgroup had the highest standardized regression coefficient value for the regression model between DI-Hg and B-Hg levels.

### Comparisons of Dietary Metal Intake

Table 4 presents overall mean dietary metal intake across the five NHANES racial/ethnic groups. Mean dietary metal intake by sociodemographic covariates for the Asian subgroups is shown in Table 5.

**Table 4:** Comparison of weighted mean dietary metal intake (μg-metal/kg-BW/day) by NHANES racial and ethnic group.

Racial/ethnic group	Sample^*a*^ size	Arsenic, total			Arsenic, inorganic			Cadmium			Lead			Mercury		
		Mean (SE) *p*-value	Percentile		Mean (SE) *p*-value	Percentile		Mean (SE) *p*-value	Percentile		Mean (SE) *p*-value	Percentile		Mean (SE) *p*-value	Percentile	
			50th	95th		50th	95th		50th	95th		50th	95th		50th	95th
Non-Hispanic Asian	785	2.00 (0.19) (ref)^*b*^	0.33	10.0	0.112 (0.003) (ref)^*b*^	0.10	0.24	0.107 (0.002) (ref)^*b*^	0.09	0.24	0.105 (0.002) (ref)^*b*^	0.09	0.22	0.086 (0.005) (ref)^*b*^	0.06	0.25
Non-Hispanic white	2,153	0.61 (0.08) < 0.001	0.15	2.41	0.049 (0.001) < 0.001	0.04	0.12	0.096 (0.003) < 0.001	0.08	0.21	0.096 (0.001) 0.004	0.09	0.20	0.058 (0.002) < 0.001	0.04	0.16
Non-Hispanic black	1,737	0.93 (0.13) < 0.001	0.14	4.91	0.049 (0.002) < 0.001	0.04	0.13	0.080 (0.002) < 0.001	0.06	0.21	0.087 (0.003) < 0.001	0.07	0.20	0.052 (0.003) < 0.001	0.03	0.16
Mexican American	788	0.74 (0.08) < 0.001	0.17	2.61	0.058 (0.002) < 0.001	0.05	0.13	0.101 (0.003) 0.09	0.09	0.22	0.107 (0.002) 0.48	0.09	0.23	0.069 (0.004) 0.005	0.05	0.21
Other Hispanic	636	0.73 (0.16) < 0.001	0.17	4.16	0.066 (0.002) < 0.001	0.05	0.16	0.088 (0.004) < 0.001	0.07	0.22	0.098 (0.004) 0.15	0.08	0.21	0.064 (0.004) 0.005	0.04	0.18
^***a***^Sample size was the same for all metals. ^***b***^Reference group.																

**Table 5:** Comparison of weighted mean dietary metal intake (μg-metal/kg-BW/day) by Asian subgroup.

Asian subgroup	Arsenic, total						Arsenic, inorganic						Cadmium						Lead						Mercury					
	C	AI	Other	*p*-Value^*b*^			C	AI	Other	*p*-Value^*b*^			C	AI	Other	*p*-Value^*b*^			C	AI	Other	*p*-Value^*b*^			C	AI	Other	*p*-Value^*b*^		
				C‑AI	C‑O	AI‑O				C‑AI	C‑O	AI‑O				C‑AI	C‑O	AI‑O				C‑AI	C‑O	AI‑O				C‑AI	C‑O	AI‑O
Overall	1.83	1.44	2.24	0.42	0.18	0.17	0.106	0.117	0.111	0.25	0.54	0.52	0.118	0.116	0.100	0.89	0.15	0.12	0.121	0.107	0.100	0.20	< 0.05*	0.38	0.084	0.095	0.083	0.54	0.84	0.42
Sex
Male	1.50	1.60	2.33	0.82	0.08	0.29	0.122	0.128	0.119	0.60	0.82	0.52	0.126	0.116	0.098	0.63	0.09	0.14	0.125	0.111	0.100	0.39	< 0.05*	0.27	0.078	0.103	0.090	0.35	0.23	0.54
Female	2.09	1.24	2.17	0.24	0.86	0.28	0.094	0.104	0.105	0.47	0.30	0.94	0.112	0.116	0.102	0.87	0.49	0.34	0.118	0.101	0.100	0.13	0.07	0.92	0.088	0.086	0.077	0.90	0.29	0.56
(*p*-Value^*a*^)	(0.19)	(0.57)	(0.75)				(< 0.05)*	(0.11)	(0.20)				(0.42)	(0.97)	(0.55)				(0.50)	(0.45)	(0.96)				(0.28)	(0.51)	(0.14)
Age
6–11 years	3.51	0.89	1.49	< 0.05*	0.06	0.24	0.194	0.204	0.178	0.72	0.48	0.27	0.271	0.236	0.162	0.47	< 0.05*	0.06	0.258	0.253	0.213	0.87	0.08	0.05	0.216	0.198	0.156	0.62	0.07	< 0.05*
12–19 years	1.38	0.39	1.58	0.27	0.86	0.13	0.080	0.138	0.089	< 0.05*	0.61	0.06	0.089	0.085	0.090	0.81	0.96	0.56	0.098	0.119	0.097	0.19	0.92	< 0.05*	0.074	0.087	0.073	0.55	0.97	0.32
20–39 years	1.87	2.28	2.02	0.75	0.79	0.87	0.099	0.099	0.104	1.00	0.78	0.76	0.099	0.099	0.101	1.00	0.91	0.82	0.111	0.094	0.090	0.32	< 0.05*	0.62	0.074	0.104	0.070	0.43	0.71	0.36
40–59 years	1.75	1.62	2.08	0.80	0.26	0.43	0.110	0.104	0.105	0.62	0.61	0.96	0.117	0.104	0.097	0.53	0.06	0.74	0.114	0.076	0.087	< 0.05*	< 0.05*	0.06	0.078	0.074	0.078	0.81	0.99	0.83
≥ 60 years	1.50	0.21	3.84	< 0.05*	< 0.05*	< 0.05*	0.096	0.108	0.126	0.71	0.14	0.58	0.119	0.108	0.084	0.60	0.11	< 0.05*	0.113	0.080	0.096	< 0.05*	0.09	0.25	0.067	0.048	0.094	0.24	0.19	< 0.05*
(*p*-Value^*a*^)	(0.42)	(< 0.05)*	(0.22)				(< 0.05)*	(< 0.05)*	(< 0.05)*				(< 0.05)*	(< 0.05)*	(< 0.05)*				(< 0.05)*	(< 0.05)*	(< 0.05)*				(< 0.05)*	(< 0.05)*	(< 0.05)*
Education
< High school (HS)	0.90	0.27	3.32	< 0.05*	< 0.05*	< 0.05*	0.097	0.095	0.138	0.84	0.05	< 0.05	0.106	0.065	0.073	< 0.05*	0.06	0.43	0.096	0.085	0.088	0.64	0.75	0.84	0.078	0.057	0.101	0.24	0.29	0.11
HS graduate/GED	1.27	4.40	1.75	0.24	0.40	0.31	0.120	0.103	0.106	0.45	0.39	0.90	0.103	0.080	0.088	0.27	0.13	0.71	0.113	0.071	0.091	< 0.05*	< 0.05*	< 0.05*	0.063	0.087	0.067	0.42	0.82	0.42
Some college/AA	1.21	1.37	1.55	0.86	0.53	0.86	0.081	0.080	0.101	0.97	0.22	0.06	0.073	0.113	0.099	0.05	0.06	0.33	0.097	0.109	0.097	0.53	0.98	0.28	0.057	0.129	0.071	0.27	0.25	0.39
≥ College graduate	2.21	1.21	2.42	0.07	0.53	< 0.05*	0.112	0.130	0.108	0.12	0.65	< 0.05	0.136	0.128	0.114	0.64	0.15	0.31	0.133	0.114	0.107	0.10	< 0.05*	0.60	0.096	0.094	0.087	0.85	0.27	0.58
(*p*-Value^*a*^)	(< 0.05)*	(0.12)	(0.11)				(0.13)	(< 0.05)*	(0.36)				(< 0.05)*	(< 0.05)*	(< 0.05)*				(0.10)	(< 0.05)*	(< 0.05)*				(< 0.05)*	(0.30)	(0.15)
Household income
< $20,000	1.30	6.45	2.13	< 0.05*	0.29	< 0.05*	0.098	0.068	0.104	< 0.05*	0.72	0.15	0.096	0.095	0.087	0.90	0.40	0.36	0.114	0.097	0.083	0.50	0.22	0.23	0.085	0.253	0.077	< 0.05*	0.70	< 0.05*
$20,000–< $50,000	2.35	0.55	1.62	< 0.05*	0.14	< 0.05*	0.107	0.119	0.122	0.63	0.35	0.90	0.104	0.110	0.093	0.83	0.63	0.24	0.098	0.108	0.093	0.67	0.61	0.43	0.077	0.068	0.076	0.68	0.96	0.58
$50,000–< $75,000	1.13	0.84	1.81	0.69	0.21	0.28	0.077	0.122	0.120	0.10	0.12	0.88	0.079	0.107	0.113	0.27	0.16	0.79	0.119	0.088	0.094	0.22	0.34	0.68	0.077	0.064	0.068	0.68	0.71	0.87
≥$75,000	1.69	1.16	2.83	0.27	0.09	< 0.05*	0.112	0.121	0.104	0.53	0.42	0.10	0.137	0.123	0.107	0.49	< 0.05*	0.36	0.136	0.115	0.112	0.18	< 0.05*	0.81	0.091	0.098	0.093	0.73	0.86	0.79
(*p*-Value^*a*^)	(0.27)	(< 0.05)*	(< 0.05)*				(0.47)	(< 0.05)*	(0.31)				(< 0.05)*	(0.25)	(0.20)				(< 0.05)*	(0.24)	(< 0.05)*				(0.79)	(< 0.05)*	(< 0.05)*
Birth place
USA	2.11	0.74	1.46	0.05	0.45	0.23	0.105	0.139	0.108	0.16	0.79	0.11	0.144	0.158	0.118	0.68	0.29	0.15	0.141	0.186	0.122	0.14	0.19	< 0.05*	0.101	0.141	0.088	0.19	0.26	0.05
Outside USA	1.73	1.59	2.61	0.80	< 0.05*	0.09	0.107	0.113	0.113	0.62	0.54	1.00	0.109	0.107	0.092	0.92	0.20	0.10	0.114	0.090	0.090	< 0.05*	< 0.05*	0.99	0.078	0.086	0.080	0.66	0.77	0.72
(*p*-Value^*a*^)	(0.50)	(0.17)	(0.16)				(0.86)	(0.24)	(0.54)				(0.19)	(< 0.05)*	(< 0.05)*				(0.10)	(< 0.05)*	(< 0.05)*				(0.14)	(< 0.05)*	(0.55)
Abbreviations: AA, Associate in Art (AA) degree; AI-Asian Indian; C, Chinese; GED, General Educational Development; O, other Asian. ^***a***^Significance of difference in mean dietary metal intake across categories within covariate. ^***b***^Significance of difference in mean dietary metal intake between each pair of Asian subgroups. *Difference is statistically significant (*p *< 0.05).																														

**Figure 2:**
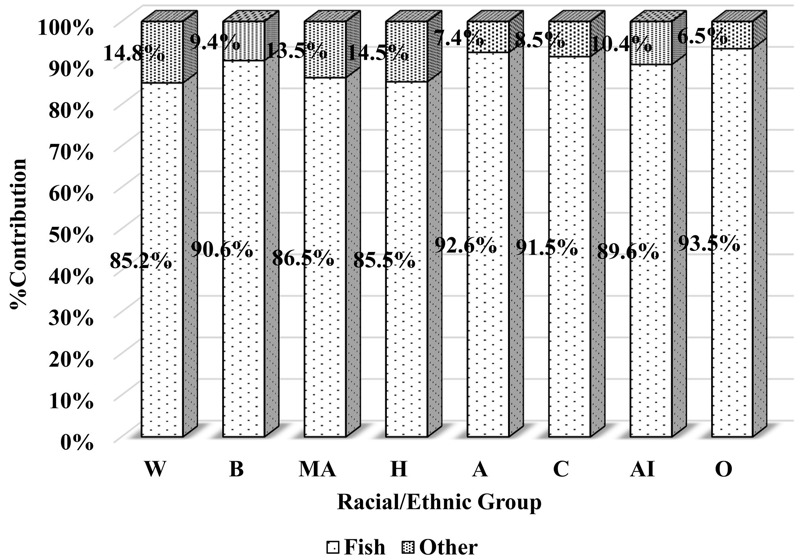
Food category–specific percent contribution to dietary arsenic (total) intake by race/ethnicity. Legend: A, Asian; AI, Asian Indian; B, black; C, Chinese; H, other Hispanic; MA, Mexican American; O, Other Asian; W, white.

***Arsenic, total.*** The Asian group had the highest overall mean DI-tAs across the five racial/ethnic groups (Table 4). In general, higher DI-tAs among the Asian group was consistently observed, independent of the various sociodemographic and geographical characteristics. DI-tAs among the Asian group was often more than twice that of other racial/ethnic groups. The majority of DI-tAs originated from fish (> 85%), regardless of racial/ethnic group. The Asian group had the highest contribution from fish (92.6%) (Figure 2). Furthermore, Asians had the highest percentage of fish consumers and the highest arithmetic mean fish consumption (see Table S1). On average, fish consumption among Asians was roughly twice that of other racial/ethnic groups.

Among the three Asian subgroups, variations in DI-tAs were minimal (Table 5). There were no apparent associations of DI-tAs with sociodemographic covariates. The observation that DI-tAs originated predominantly from fish did not vary across Asian subgroups. However, substantial variations in fish consumption patterns did exist among these subgroups (see Table S2). The Asian-Indian subgroup had a considerably lower percentage of fish consumers and a lower average fish consumption, compared with the Chinese and Other Asian subgroups.

**Figure 3:**
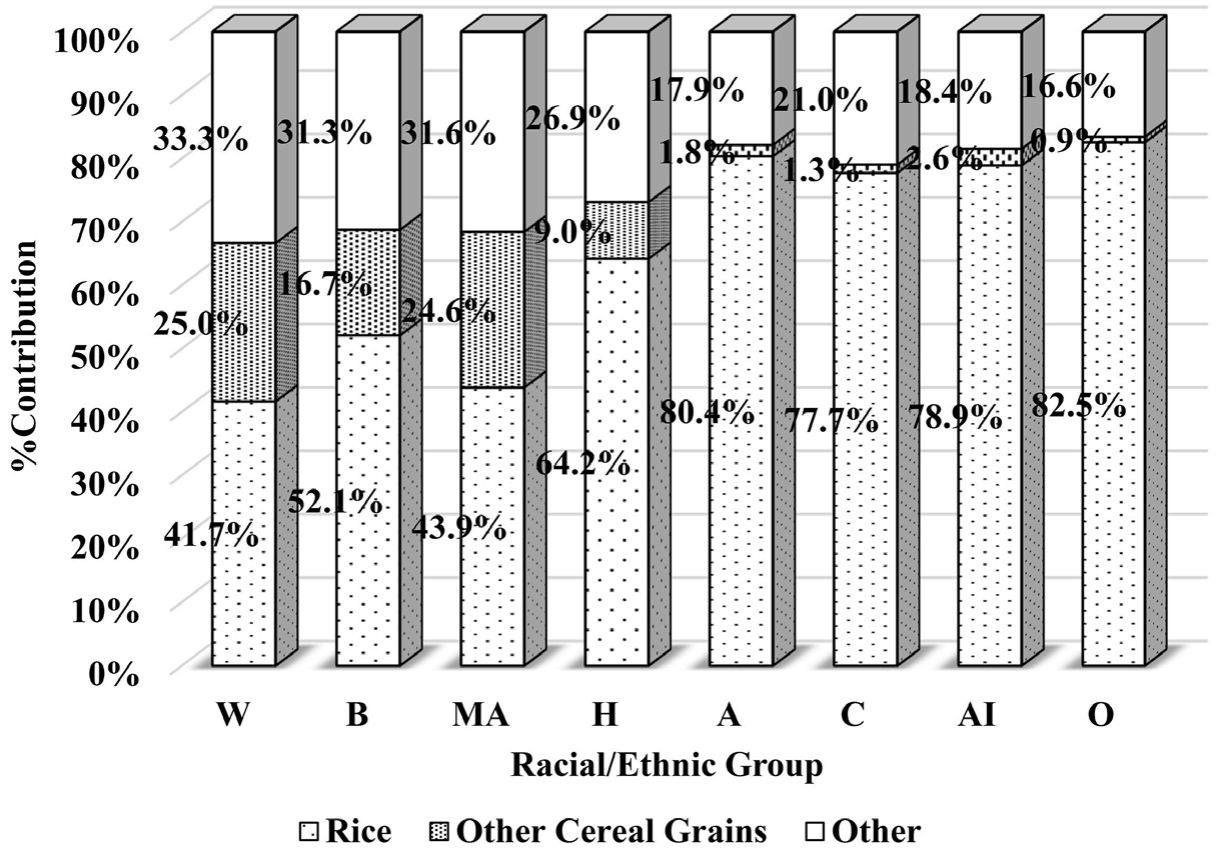
Food category–specific percent contribution to dietary arsenic (inorganic) intake by race/ethnicity. Legend: A, Asian; AI, Asian Indian; B, black; C, Chinese; H, other Hispanic; MA, Mexican American; O, Other Asian; W, white.

***Arsenic, inorganic.*** Similar to the DI-tAs results, the overall mean DI-iAs was highest among the Asian group (Table 4). Also, Asians had significantly higher DI-iAs as compared with all other racial/ethnic groups in nearly all of the comparisons performed within the sociodemographic and geographic covariate categories. The contribution from cereal grains was the highest across different food categories, ranging from 67% (white) to 82.1% (Asians) (Figure 3). Rice made up most of the DI-iAs from cereal grains among Asians, whereas the contribution of rice to overall DI-iAs from cereal grains was lower among other racial/ethnic groups. Asians consumed more rice (white and brown) than any other racial/ethnic group, in terms of rice consumption percentage and mean rice consumption (see Table S1).

DI-iAs was similar among Asian subgroups. There was no apparent pattern of differences in DI-iAs based on the sociodemographic covariates, except by age groups, where there were some significant differences. Children (6–11 years) had the highest DI-iAs but no noticeable differences were observed across the next four older age groups. Unlike the fish consumption results, no apparent differences in rice consumption was observed across the three Asian subgroups (see Table S2).

***Cadmium.*** Asians had the highest overall mean DI-Cd (Table 4). Vegetables were the largest source of DI-Cd, accounting for > 56% of the total DI-Cd, followed by cereal grains (~ 15–20%), fruits (~ 6–8%), and dairy (~ 5–6%) (see Figure S1). Although the general makeup of DI-Cd sources was similar across the racial/ethnic groups, the contribution of rice to overall DI-Cd from cereal grains among Asians was two to six times higher than those of other racial/ethnic groups.

There was little variation in DI-Cd across the Asian subgroups (Table 5). A similar pattern of age-related differences that was observed for DI-iAs was also seen in DI-Cd. There was a trend toward increasing DI-Cd levels with greater educational status in all three Asian subgroups. Asian Indians and Other Asians born outside the United States had significantly lower DI-Cd than those born in the United States. The source of DI-Cd, was similar across the Asian subgroups (see Figure S1).

***Lead.*** Overall DI-Pb was highest among Mexican-Americans, but not significantly higher than among Asians (Table 4). The degree and statistical significance of difference in DI-Pb between Asians and other racial/ethnic groups were the least of the five metals. DI-Pb was more widely distributed among different food categories than other metals. The four largest DI-Pb contributors were vegetables, fruits, cereal grains, and dairy, with each of these contributing 14–24% of total DI-Pb, depending on the racial/ethnic group (see Figure S2).

As was the case with DI-Cd, there was little variation in DI-Pb among Asian subgroups (Table 5). The patterns of the association of DI-Pb with age, education, and birthplace were similar to those observed for DI-Cd. Further, the sources of DI-Pb were similar across Asian subgroups, except for a higher DI-Pb contribution from dairy sources among Asian-Indians (see Figure S2).

***Mercury.*** Although chemical data on mercury in the TDS were limited due to their specific data collection focus on fish and other seafood, we also estimated DI-Hg. Asians had significantly higher overall mean DI-Hg than the other main racial/ethnic groups (Table 4); however, the differences were not as pronounced as those seen in DI-tAs with regard to their degree and statistical significance. Dairy was the highest source of DI-Hg for whites, Mexican Americans, and other Hispanics, accounting for approximately 34–41% of DI-Hg. Among blacks and Asians, however, the largest DI-Hg contribution was from fish (see Figure S3).

As with other metals, variations in DI-Hg across the three Asian subgroups were minimal (Table 5). There was no apparent association of DI-Hg with sociodemographic covariates, except for age-related differences similar to those observed for DI-iAs, DI-Cd, and DI-Pb. Moreover, a higher DI-Hg contribution from fish was observed among the Other Asian subgroup, whereas there was generally little difference in the sources of DI-Hg between Chinese and Indian Asian subgroups (see Figure S3).

## Discussion

The daily food consumption and dietary metal intake estimated in our study were in general agreement with the results presented in previous studies. We estimated a mean daily consumption of seafood ranging from 0.36 (white) to 0.84 g/kg/day (Asians). The range of the mean per capita consumption of seafood (finfish and shellfish combined) based on the analysis of the 2003–2006 NHANES data (all ages) by the U.S. EPA was 0.23 (Mexican American) to 0.45 g/kg/day for the “other” ethnic group including Asians (U.S. EPA 2011). Additionally, the study of adults’ seafood consumption (≥ 18 years old) from 10 Asian American and Pacific Islander ethnic groups in King County, Washington, estimated the mean consumption of all seafood to be 1.89 g/kg/day (Sechena et al. 1999). Furthermore, the estimated daily consumption of rice in our study was 0.27 (white) to 1.23 g/kg/day (Asians). The U.S. EPA estimated the per capita consumption of rice to be between 0.2 (white) and 0.8 g/kg/day for the “other” ethnic group based on the 2003–2006 NHANES data (U.S. EPA 2011).

Further, the dietary metal intake levels estimated in the present study generally agreed with the results presented previously. Xue et al. (2010) computed Di-tAs and Di-iAs based on NHANES 2003–2004 using a probabilistic exposure model. The ranges of the estimated mean DI-tAs and DI-iAs in various age groups ≥ 6 years of age were 0.25–0.37 and 0.03–0.05 μg/kg/day, respectively. Our estimations of the mean DI-tAs and DI-iAs were slightly higher than those estimated by Xue et al. (2010), with respective mean intake ranges of 0.61–2.0 and 0.05–0.11 μg/kg/day. An average DI-Cd based on the national representative food consumption data (ages ≥ 1 year) in the United States between 1989 and 1991 was 0.2 μg/kg/day (Dougherty et al. 2000). We estimated DI-Cd to be 0.08–0.11 μg/kg/day in our study. A probabilistic analysis of DI-Hg based on NHANES 1999–2006 estimated DI-Hg across different age groups ≥ 6 years of age to be 0.01–0.05 μg/kg/day for the “other” race group including Asians and to be 0.01–0.02 μg/kg/day for the rest of racial/ethnic groups combined (white, black, and Mexican American) (Xue et al. 2012). Our estimated mean DI-Hg was 0.09 μg/kg/day for Asians and 0.05–0.07 μg/kg/day for the rest of the four racial/ethnic groups. The estimated DI-Pb previously reported was similar to the levels estimated in our study. The estimated mean DI-Pb among population-based samples from the EPA Region V (Midwest states including Indiana, Illinois, Michigan, Minnesota, Ohio, and Wisconsin) by Thomas et al. (1999) was 0.25 μg/kg/day. We estimated the average DI-Pb to be approximately 0.1 μg/kg/day.

Using nationally representative data, our study confirmed that DI-tAs, DI-iAs, and DI-Hg are key pathways of arsenic and mercury exposures and are significantly associated with their corresponding biomarker levels among the Asian populations in the United States. Despite the high fish consumption rate in the Chinese subgroup, the regression model for total arsenic in this subgroup had the lowest standardized regression coefficient and highest *p*-value, suggesting that there may be other arsenic exposure sources or different levels of confounding (e.g., smoking) in this subgroup. The standardized regression coefficients for arsenic (total and inorganic) among Asians were greater than those of other racial/ethnic groups, suggesting that other factors, which differ across racial/ethnic groups, may influence these associations (e.g., more efficient absorption, poorer elimination). In comparison to other metals, the difference in the estimated DI-tAs and DI-iAs between Asians and the other racial/ethnic groups was greater (often two times higher) and statistically significant. The significant difference was most pronounced for DI-iAs. We also confirmed that fish (for total arsenic and mercury) and rice (for inorganic arsenic) are the predominant contributors to the dietary intake of metals among Asians. Previously, this had only been inferred from the data of the aggregated race group (i.e., “other” racial group in the NHANES which was comprised of small minority populations such as Native Americans, Pacific Islanders, and multiple racial individuals) (Wei et al. 2014; Xue et al. 2012). Although arsenic consumed through fish is considered to be mostly the less harmful organic forms of arsenic, the higher DI-tAs in Asians are worth noting.

Unlike arsenic exposure, there was no compelling evidence that estimated dietary intake is an important exposure pathway for cadmium and lead exposure among Asians. Although the estimated DI-Cd was generally the highest within the Asian population, no significant association between B-Cd level and DI-Cd was observed. There appeared to be less evidence supporting the hypothesis that DI-Pb contributed to B-Pb among Asians. Although not always significant, Mexican Americans had higher mean DI-Pb than Asians. DI-Pb was not a significant predictor of B-Pb levels among Asians, but it was among Mexican Americans. These results suggest that contributions from nondietary sources may be important for cadmium and lead exposures among Asian populations, which is consistent with our understanding of the exposure characteristics of cadmium and lead in the general U.S. population (ATSDR 2007b, 2012). Smoking is the main exposure route for cadmium, followed by food consumption. Likewise, exposure to lead can originate from various environmental and occupational sources. Adjusting data for these exposure sources may have improved our evaluation of dietary contributions.

Aside from these findings, there is another important consideration when interpreting the results: The metals evaluated in our study have different half-lives and toxicokinetics characteristics in the human body. For instance, cadmium in blood exhibits the first component of elimination with a half-life of 3–4 months, followed by a slow component with a half-life of 10 years (Järup et al. 1983); therefore, B-Cd may be a reflective of body burden from long-term exposure. On the other hand, arsenic has a shorter half-life (~ 2–3 days), and its biomarker levels may be a better representation of short-term exposure (ATSDR 2015). This may be another reason we observed a positive relationship between the U-tAs and U-DMA levels and their estimated dietary levels, because the dietary data we used were obtained from 24-hr recall rather than long-term food consumption surveys.

The limitations associated with the present study stemmed mainly from two sources: the estimation of metal concentrations in food and the application of the NHANES food consumption data. The estimation of metal concentrations has some limitations. Concentrations in food commodities were estimated based on a single representative concentration and did not account for variations in chemical concentrations across different food types, geographical locations of cultivation (Meharg et al. 2009; Williams et al. 2005, 2007), and growing methods (Barański et al. 2014), among other factors within a single food commodity. For instance, the commodity “saltwater fish” includes a wide variety of fish species that can have different mercury content (FDA 2014b). Additionally, we were not able to assign chemical data to all of the target food commodities due to a lack of data. Seaweed is a good example; it may have an elevated metal content (Almela et al. 2006; Rose et al. 2007) and can be an important source of dietary metal intake (Lee et al. 2006). Therefore, omission of such a food commodity in the estimation of metal concentrations will underestimate overall dietary metal intake. Further, the composition of food commodities was assumed to be the same, although there may be variations in food recipe and preparations. Moreover, we used an LOD-based “fill value” for the nondetected results in the estimation of metal concentrations in food. The use of the fill value may have diluted the importance of food with high metal contents in the estimation of overall dietary intake and likely weakened the association between biomarker levels and dietary metal intake, especially for mercury. Also, there may be some uncertainties associated with the use of inorganic arsenic data from the study by Schoof et al. which were collected in an older time period (i.e., 1997).

Other limitations of the study are attributable to the NHANES consumption data. We estimated the daily amount of food consumed, based on 24-hr recall dietary data. Therefore, the data may only represent a snapshot of study participants’ dietary consumption and may not reflect their long-term food consumption patterns. In addition, these data are subject to recall bias. Further, the relatively small sample sizes of Asian subgroups from one data cycle may have produced statistically unreliable results that should be viewed with caution. The results of our study should be verified based on a larger data set, appending the data from the continuous oversampling of Asian populations in the 2012–2014 NHANES in the future.

The major strengths of this study are attributed to use of national representative data of Asian populations from NHANES. We believe that this is one of the first works to investigate the dietary consumption and dietary metal intake of Asians on a national scale. Currently, studies of food consumption and dietary metal intake in Asian population in the United States are limited mainly to those ethnic groups in Far East Asia (such as Chinese, Japanese, and Koreans) and to cohorts from geographic areas with high Asian populations (e.g., New York City, Hawaii, and California). As fractions of Asian ethnic groups (e.g., Asian Indians) originating from regions other than the Far East are becoming larger, and residences of Asians in the United States have become more geographically diverse in the past decade (U.S. Census Bureau 2013), our study provides more comprehensive characteristics of Asian populations in the United States than previous studies. Other advantages of use of the NHANES data are its relatively large sample size and ability to account for study participants’ various sociodemographic and geographic characteristics in our data analysis. Furthermore, our study evaluated comprehensive dietary metal intake estimated based on a large number of target food commodities, rather than food consumption or consumption frequency of limited food items that were often used as bases in the previous studies.

## Conclusion

To our knowledge, this is one of the first studies evaluating dietary intake as a potential cause of the elevated biomarker levels of four metals (arsenic, cadmium, lead, and mercury) previously seen among Asian populations in the United States. We confirmed that dietary intake is an important exposure pathway for total and inorganic arsenic and mercury. Our study also confirmed that fish (for total arsenic and mercury) and rice (for inorganic arsenic) are important dietary sources of their arsenic and mercury exposures. The results of cadmium and lead were not as conclusive as those of arsenic and mercury, indicating contributions from nondietary sources may be important for cadmium and lead.

## Supplemental Material

(520 KB) PDF
