# Supplementary material for: Association of Dietary Intake and Biomarker Levels of Arsenic, Cadmium, Lead, and Mercury among Asian Populations in the United States: NHANES 2011–2012
Source: Environ Health Perspect. 2016 Sep 2;125(3):314–23. doi: 10.1289/EHP28 (PMC5332183; doi:10.1289/EHP28)
Supplement: (520 KB) PDF [file EHP28.s001.acco.pdf]

*Environ Health Perspect*
DOI: 10.1289/EHP28

**Note to readers with disabilities:** *EHP* strives to ensure that all journal content is accessible to all readers. However, some figures and Supplemental Material published in *EHP* articles may not conform to 508 standards due to the complexity of the information being presented. If you need assistance accessing journal content, please contact ehp508@niehs.nih.gov. Our staff will work with you to assess and meet your accessibility needs within 3 working days.

# Supplemental Material

# Association of Dietary Intake and Biomarker Levels of Arsenic, Cadmium, Lead, and Mercury among Asian Populations in the U.S.: NHANES 2011–2012

Hiroshi Awata, Stephen Linder, Laura E. Mitchell, and George L. Delclos

## Table of Contents

**Table S1.** Per Capita Intake of Food (g-food/kg-BW/day) Across Race and Ethnic Groups

**Table S2.** Per Capita Intake of Food (g-food/kg-BW/day) Across Asian Subgroups

**Figure S1.** Food-category-specific %contribution to dietary cadmium intake by race/ethnicity

**Figure S2.** Food-category-specific %contribution to dietary lead intake by race/ethnicity

**Figure S3.** Food-category-specific %contribution to dietary mercury intake by race/ethnicity

Table S1 - Per Capita Intake of Food (g-food/kg-BW/day) Across Race and Ethnic Groups

| | Non-Hispanic White N=2153 | | | | | Non-Hispanic Black N=1738 | | | | | Mexican American N=788 | | | | | Other Hispanic N=636 | | | | | Non-Hispanic Asian N=785 | | | | |
|---|---|---|---|---|---|---|---|---|---|---|---|---|---|---|---|---|---|---|---|---|---|---|---|---|---|
| Food Category | (%)[a] | Mean | SE | Percentile 50th | Percentile 95th | (%)[a] | Mean | SE | Percentile 50th | Percentile 95th | (%)[a] | Mean | SE | Percentile 50th | Percentile 95th | (%)[a] | Mean | SE | Percentile 50th | Percentile 95th | (%)[a] | Mean | SE | Percentile 50th | Percentile 95th |
| Vegetables | (100.0) | 3.69 | 0.123 | 3.06 | 8.30 | (100.0) | 3.07 | 0.080 | 2.41 | 7.66 | (100.0) | 3.55 | 0.080 | 3.05 | 7.52 | (100.0) | 3.41 | 0.168 | 2.75 | 8.13 | (100.0) | 4.63 | 0.115 | 4.14 | 10.16 |
| Fruits | (98.6) | 3.00 | 0.124 | 2.04 | 9.05 | (98.7) | 2.85 | 0.108 | 1.81 | 9.58 | (98.3) | 3.51 | 0.172 | 2.28 | 12.08 | (98.4) | 3.70 | 0.238 | 2.53 | 11.18 | (99.4) | 3.90 | 0.229 | 2.80 | 11.39 |
| Mushroom | (19.9) | 0.05 | 0.005 | -- | 0.31 | (11.6) | 0.03 | 0.004 | -- | 0.18 | (14.6) | 0.03 | 0.006 | -- | 0.18 | (13.1) | 0.04 | 0.007 | -- | 0.24 | (32.0) | 0.11 | 0.016 | -- | 0.57 |
| Nuts | (59.4) | 0.12 | 0.014 | <0.01 | 0.61 | (52.2) | 0.07 | 0.011 | <0.01 | 0.31 | (54.3) | 0.07 | 0.008 | <0.01 | 0.33 | (47.5) | 0.16 | 0.073 | -- | 0.34 | (64.7) | 0.24 | 0.040 | 0.02 | 0.87 |
| Herbs/Spices | (98.5) | 0.01 | 0.001 | 0.01 | 0.03 | (96.7) | 0.01 | 0.001 | 0.01 | 0.04 | (95.5) | 0.02 | 0.001 | 0.01 | 0.04 | (93.8) | 0.02 | 0.002 | 0.01 | 0.05 | (94.5) | 0.01 | 0.001 | 0.01 | 0.04 |
| Cereal Grains | (100.0) | 2.98 | 0.055 | 2.53 | 6.55 | (100.0) | 2.97 | 0.090 | 2.37 | 7.15 | (100.0) | 3.60 | 0.126 | 3.26 | 7.55 | (99.2) | 3.11 | 0.151 | 2.64 | 7.07 | (100.0) | 3.40 | 0.080 | 3.07 | 6.77 |
| Rice, white | (42.8) | 0.23 | 0.016 | -- | 1.01 | (44.5) | 0.29 | 0.029 | -- | 1.29 | (52.9) | 0.32 | 0.022 | 0.02 | 1.36 | (70.8) | 0.53 | 0.048 | 0.42 | 1.71 | (84.3) | 1.09 | 0.034 | 0.97 | 2.73 |
| Rice, brown | (12.2) | 0.04 | 0.006 | -- | 0.22 | (8.4) | 0.05 | 0.010 | -- | 0.28 | (8.7) | 0.03 | 0.007 | -- | 0.08 | (9.6) | 0.05 | 0.012 | -- | 0.47 | (20.4) | 0.14 | 0.022 | -- | 0.90 |
| Beef | (86.4) | 0.85 | 0.044 | 0.63 | 2.62 | (86.9) | 0.80 | 0.071 | 0.51 | 2.56 | (86.5) | 1.10 | 0.093 | 0.78 | 3.40 | (79.0) | 0.84 | 0.062 | 0.55 | 2.60 | (76.8) | 0.79 | 0.053 | 0.34 | 3.00 |
| Pork | (79.6) | 0.43 | 0.023 | 0.21 | 1.54 | (80.7) | 0.45 | 0.040 | 0.24 | 1.67 | (85.5) | 0.44 | 0.021 | 0.28 | 1.29 | (78.1) | 0.43 | 0.033 | 0.23 | 1.56 | (71.3) | 0.56 | 0.055 | 0.15 | 2.08 |
| Poultry | (72.4) | 0.93 | 0.031 | 0.75 | 3.00 | (84.3) | 1.25 | 0.031 | 1.03 | 3.55 | (76.4) | 1.09 | 0.049 | 0.80 | 3.19 | (76.2) | 1.26 | 0.092 | 1.00 | 3.72 | (82.2) | 1.23 | 0.074 | 0.98 | 3.87 |
| Other Meat | (13.9) | 0.02 | 0.004 | -- | 0.01 | (21.3) | 0.02 | 0.010 | -- | 0.02 | (12.2) | 0.02 | 0.014 | -- | 0.01 | (12.8) | 0.03 | 0.018 | -- | 0.01 | (16.8) | 0.04 | 0.008 | -- | 0.02 |
| Fish | (28.9) | 0.36 | 0.046 | -- | 1.94 | (32.9) | 0.47 | 0.063 | -- | 2.54 | (26.9) | 0.43 | 0.052 | -- | 2.80 | (28.3) | 0.43 | 0.075 | -- | 2.61 | (52.9) | 0.84 | 0.058 | 0.01 | 3.52 |
| Fish-freshwater | (6.2) | 0.07 | 0.015 | -- | 0.03 | (7.4) | 0.11 | 0.023 | -- | 0.60 | (2.9) | 0.05 | 0.019 | -- | -- | (8.1) | 0.08 | 0.030 | -- | 0.05 | (16.5) | 0.15 | 0.027 | -- | 1.37 |
| Fish-saltwater | (18.1) | 0.20 | 0.032 | -- | 1.37 | (20.0) | 0.29 | 0.039 | -- | 1.80 | (16.8) | 0.27 | 0.049 | -- | 1.81 | (16.7) | 0.23 | 0.055 | -- | 1.53 | (40.5) | 0.58 | 0.062 | -- | 3.09 |
| Fish-shellfish | (11.4) | 0.12 | 0.016 | -- | 0.79 | (13.8) | 0.11 | 0.021 | -- | 0.77 | (13.2) | 0.13 | 0.033 | -- | 0.71 | (12.6) | 0.14 | 0.040 | -- | 0.92 | (27.3) | 0.25 | 0.038 | -- | 1.46 |
| Dairy | (99.9) | 4.17 | 0.134 | 2.63 | 13.66 | (99.8) | 2.69 | 0.216 | 1.36 | 9.98 | (100.0) | 4.70 | 0.203 | 3.07 | 14.60 | (99.9) | 4.04 | 0.192 | 2.46 | 12.11 | (99.2) | 3.85 | 0.207 | 2.09 | 13.72 |
| Egg | (95.3) | 0.38 | 0.017 | 0.17 | 1.40 | (96.8) | 0.38 | 0.019 | 0.19 | 1.43 | (94.8) | 0.57 | 0.033 | 0.30 | 1.87 | (93.3) | 0.50 | 0.039 | 0.19 | 1.83 | (90.8) | 0.47 | 0.017 | 0.26 | 1.67 |
| Oil | (99.9) | 0.06 | 0.002 | 0.04 | 0.18 | (100.0) | 0.06 | 0.003 | 0.04 | 0.19 | (100.0) | 0.07 | 0.006 | 0.04 | 0.23 | (99.2) | 0.04 | 0.003 | 0.03 | 0.12 | (99.9) | 0.05 | 0.003 | 0.03 | 0.14 |

Units are presented in g-food/kg-body weight/day. [a] Individuals who consumed any food commodities within food category were identified as Consumer. -- Not calculated due to high frequency of non-detected results (below limit of detection). SE - Standard Error, BW- Body Weight.

Table S2 - Per Capita Intake of Food (g-food/kg-BW/day) Across Asian Subgroups

| Food Category | Chinese | | | | | Asian Indian | | | | | Other Asian | | | | |
|---|---|---|---|---|---|---|---|---|---|---|---|---|---|---|---|
| | (%)[a] | Mean | SE | Percentile | | (%)[a] | Mean | SE | Percentile | | (%)[a] | Mean | SE | Percentile | |
| | | | | 50th | 95th | | | | 50th | 95th | | | | 50th | 95th |
| Vegetables | (100.0) | 5.62 | 0.316 | 5.22 | 10.45 | (100.0) | 4.63 | 0.344 | 4.19 | 8.37 | (100.0) | 4.35 | 0.152 | 3.65 | 10.21 |
| Fruits | (99.4) | 5.20 | 0.564 | 4.40 | 12.28 | (100.0) | 3.72 | 0.424 | 2.76 | 9.57 | (99.2) | 3.59 | 0.260 | 2.71 | 11.06 |
| Mushroom | (45.0) | 0.15 | 0.028 | -- | 0.67 | (25.9) | 0.06 | 0.013 | -- | 0.28 | (30.5) | 0.12 | 0.023 | -- | 0.57 |
| Nuts | (48.6) | 0.10 | 0.016 | -- | 0.52 | (76.2) | 0.23 | 0.049 | 0.08 | 0.82 | (65.3) | 0.28 | 0.053 | 0.02 | 1.25 |
| Herbs/Spices | (91.3) | 0.01 | 0.002 | 0.01 | 0.05 | (95.7) | 0.01 | 0.002 | 0.01 | 0.06 | (94.9) | 0.01 | 0.001 | 0.01 | 0.03 |
| Cereal Grains | (100.0) | 3.16 | 0.176 | 2.80 | 5.65 | (100.0) | 3.65 | 0.173 | 3.31 | 7.48 | (100.0) | 3.38 | 0.107 | 3.07 | 6.77 |
| Rice, white | (90.8) | 1.03 | 0.067 | 0.99 | 2.20 | (77.5) | 1.09 | 0.091 | 0.96 | 2.75 | (84.9) | 1.11 | 0.062 | 0.95 | 2.79 |
| Rice, brown | (17.4) | 0.09 | 0.051 | -- | 0.36 | (27.4) | 0.16 | 0.028 | -- | 1.33 | (18.8) | 0.15 | 0.029 | -- | 0.84 |
| Beef | (77.7) | 0.90 | 0.130 | 0.50 | 2.98 | (59.6) | 0.34 | 0.058 | <0.01 | 2.08 | (82.6) | 0.92 | 0.095 | 0.56 | 3.36 |
| Pork | (83.1) | 0.95 | 0.115 | 0.72 | 2.63 | (56.4) | 0.07 | 0.010 | <0.01 | 0.35 | (73.2) | 0.61 | 0.064 | 0.23 | 2.05 |
| Poultry | (79.6) | 1.18 | 0.161 | 0.97 | 3.59 | (69.7) | 1.05 | 0.187 | 0.50 | 3.53 | (87.3) | 1.30 | 0.064 | 1.11 | 3.96 |
| Other Meat | (20.1) | 0.01 | 0.012 | -- | 0.02 | (10.1) | 0.08 | 0.033 | -- | 0.18 | (18.2) | 0.03 | 0.008 | -- | 0.01 |
| Fish | (59.4) | 0.86 | 0.107 | 0.29 | 3.59 | (31.4) | 0.51 | 0.166 | -- | 3.04 | (58.7) | 0.95 | 0.085 | 0.27 | 3.51 |
| Fish-freshwater | (19.5) | 0.15 | 0.036 | -- | 1.40 | (11.9) | 0.08 | 0.040 | -- | 0.52 | (17.3) | 0.17 | 0.040 | -- | 1.38 |
| Fish-saltwater | (44.9) | 0.55 | 0.067 | -- | 3.24 | (24.8) | 0.43 | 0.170 | -- | 3.04 | (44.7) | 0.63 | 0.071 | -- | 3.07 |
| Fish-shellfish | (38.5) | 0.35 | 0.105 | -- | 1.71 | (11.1) | 0.03 | 0.014 | -- | 0.19 | (29.8) | 0.30 | 0.047 | -- | 1.56 |
| Dairy | (100.0) | 4.14 | 0.452 | 2.71 | 11.61 | (98.4) | 5.83 | 0.622 | 3.51 | 20.23 | (99.3) | 3.07 | 0.217 | 1.73 | 11.69 |
| Egg | (95.4) | 0.66 | 0.061 | 0.34 | 2.07 | (81.4) | 0.37 | 0.052 | 0.14 | 1.31 | (92.8) | 0.45 | 0.013 | 0.25 | 1.43 |
| Oil | (100.0) | 0.06 | 0.008 | 0.04 | 0.18 | (100.0) | 0.04 | 0.005 | 0.03 | 0.11 | (99.8) | 0.05 | 0.003 | 0.03 | 0.14 |

Units are presented in g-food/kg-body weight/day. [a] Individuals who consumed any food commodities within food category were identified as Consumer. -- Not calculated due to high frequency of non-detected results (below limit of detection). SE - Standard Error, BW- Body Weight.

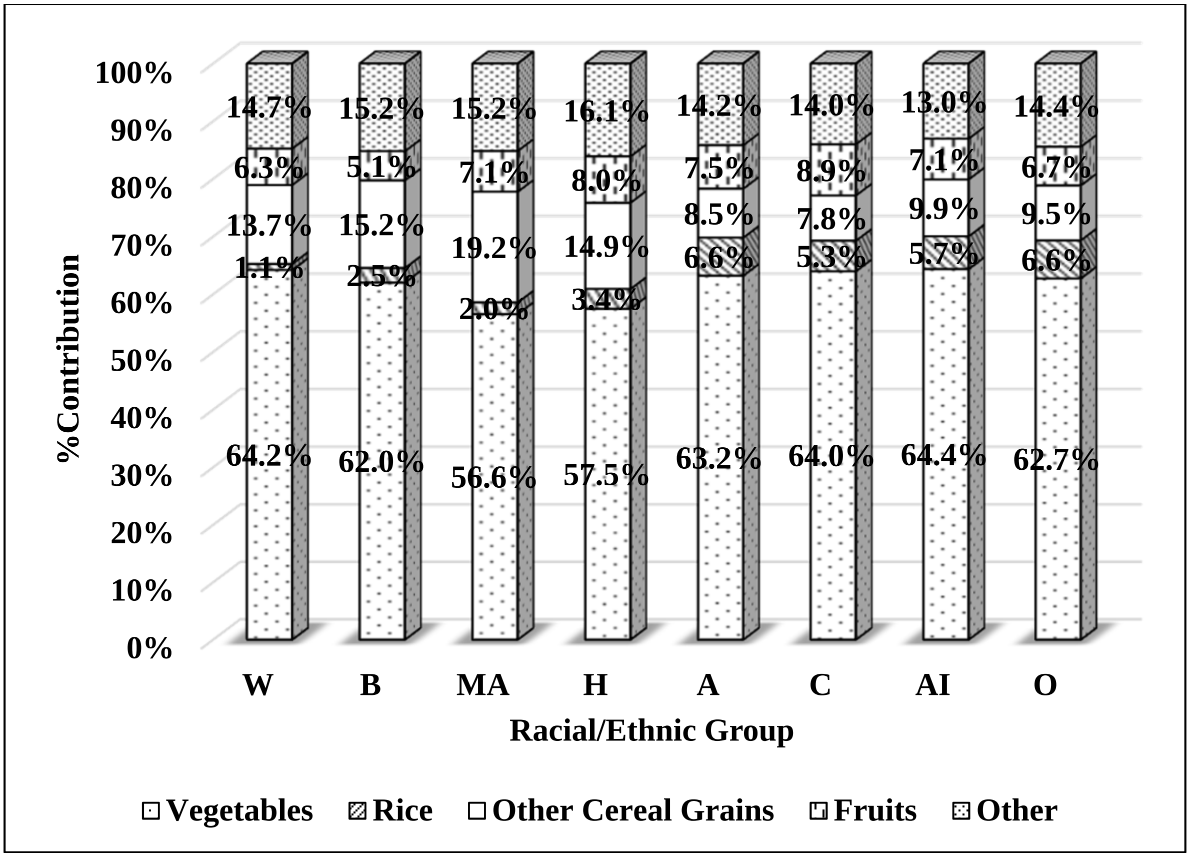


**Figure S1. Food-category-specific %contribution to dietary cadmium intake by race/ethnicity**

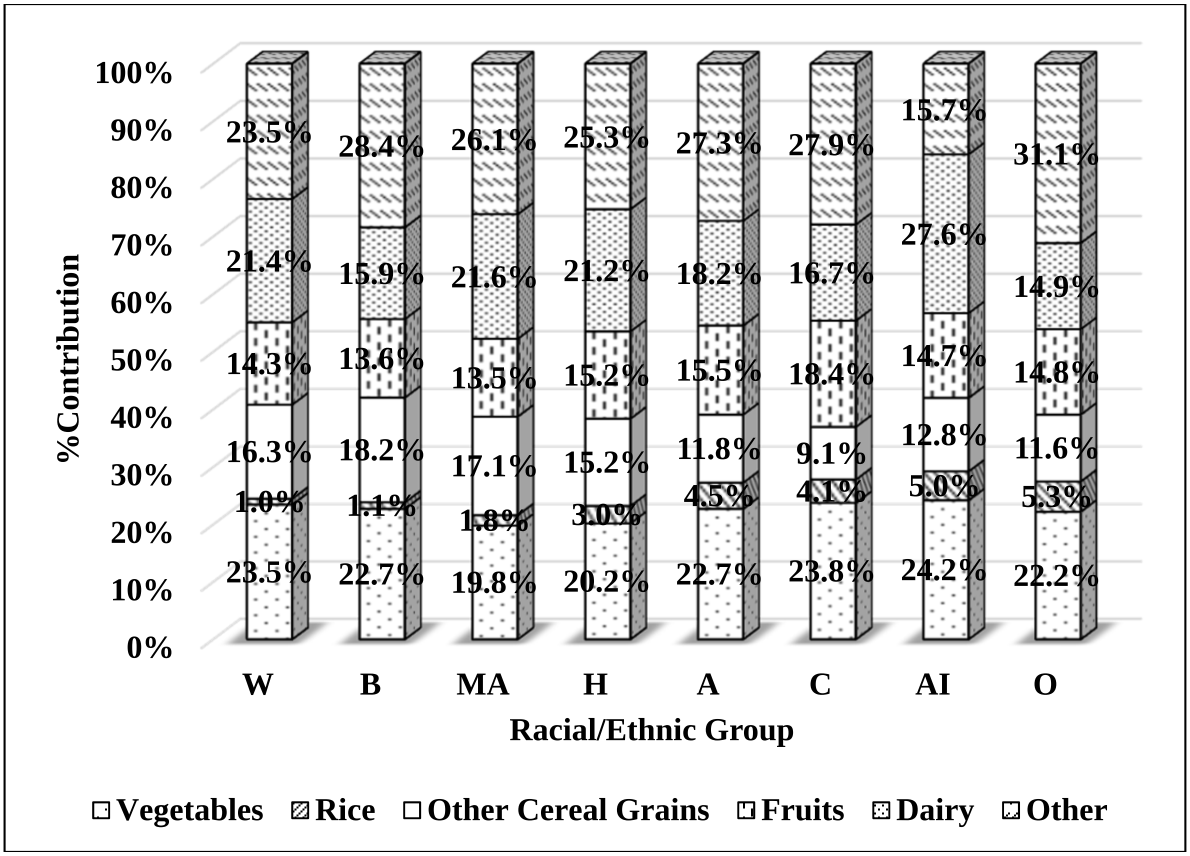


**Figure S2. Food-category-specific %contribution to dietary lead intake by race/ethnicity**

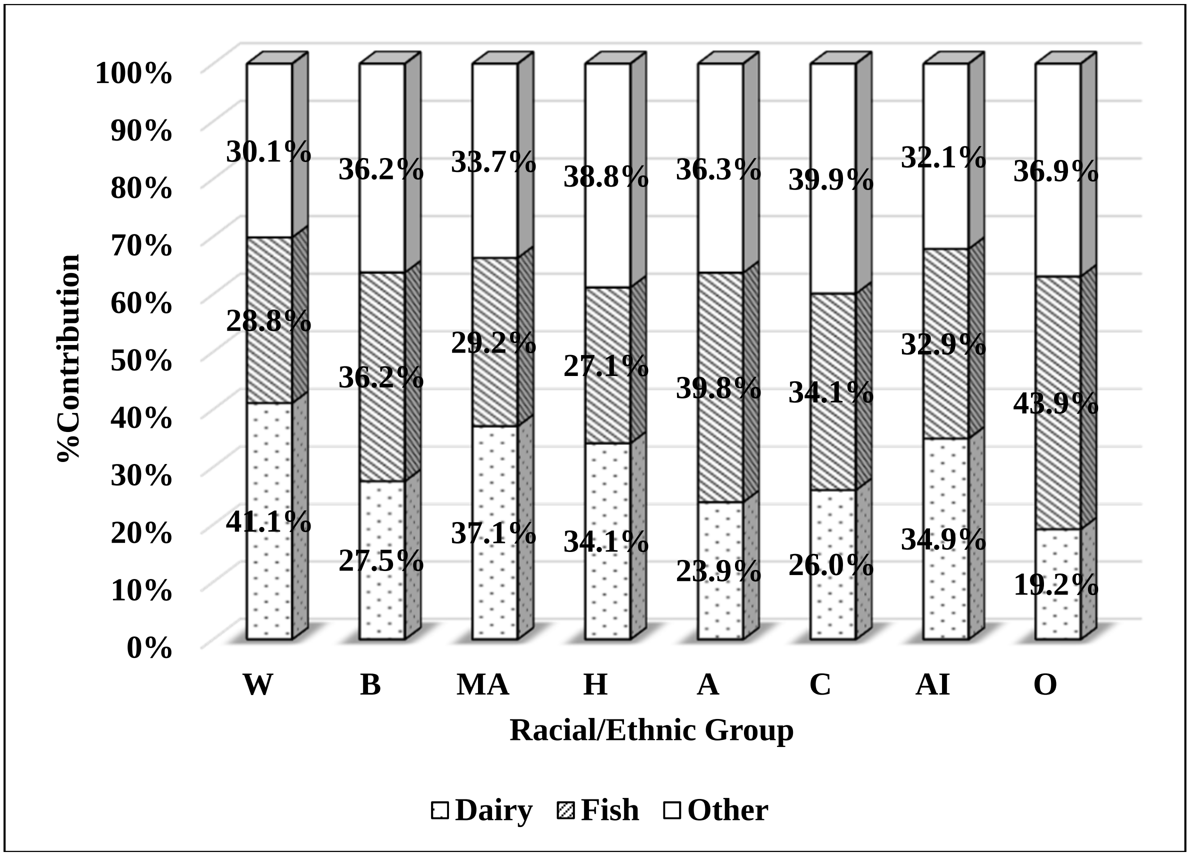


**Figure S3. Food-category-specific %contribution to dietary mercury intake by race/ethnicity**
